# Effects of Human Umbilical Cord-Derived Mesenchymal Stem Cells on the Acute Cigarette Smoke-Induced Pulmonary Inflammation Model

**DOI:** 10.3389/fphys.2020.00962

**Published:** 2020-08-12

**Authors:** Xiao-Yue Chen, Yi-Ying Chen, Willie Lin, Chia-Wen Chien, Chien-Han Chen, Yu-Chieh Wen, Ta-Chih Hsiao, Hsiao-Chi Chuang

**Affiliations:** ^1^School of Respiratory Therapy, College of Medicine, Taipei Medical University, Taipei, Taiwan; ^2^Meridigen Biotech Co. Ltd., Taipei, Taiwan; ^3^Graduate Institute of Environmental Engineering, National Taiwan University, Taipei, Taiwan; ^4^Cell Physiology and Molecular Image Research Center, Wan Fang Hospital, Taipei Medical University, Taipei, Taiwan; ^5^Division of Pulmonary Medicine, Department of Internal Medicine, Shuang Ho Hospital, Taipei Medical University, New Taipei City, Taiwan

**Keywords:** cigarette smoke, inflammation, mesenchymal stem cells, oxidative stress, lung

## Abstract

Cigarette smoke (CS) has been reported to induce oxidative stress and inflammatory process in the lungs. However, the role of human umbilical cord-derived mesenchymal stem cells (hUC-MSCs) in the regulation of pulmonary inflammation remains unclear. The objective of this study is to investigate the effects of hUC-MSCs on lung inflammation in the acute CS-induced pulmonary inflammation animal model. Eight-week-old male C57BL/6 mice were intravenously administered 3 × 10^6^, 1 × 10^7^, and 3 × 10^7^ cells/kg of hUC-MSCs as well as normal saline alone (control) after 3 days of CS exposure. Mice exposed to high-efficiency particulate air (HEPA)-filtered room air served as the CS control group. High-dose (3 × 10^7^ cells/kg) hUC-MSC administration significantly decreased tumor necrosis factor (TNF)-α in the bronchoalveolar lavage fluid (BALF) of CS-exposed mice (*p* < 0.05). The chemokine (CXC motif) ligand 1/keratinocyte chemoattractant (CXCL1/KC) in BALF were significantly reduced by low-dose (3 × 10^6^ cells/kg) and high-dose (3 × 10^7^ cells/kg) hUC-MSC (*p* < 0.05). Medium-dose hUC-MSC administration decreased interleukin (IL)-1β in lung of mice, and TNF-α and caspase-3 were decreased in the lung of CS-exposed mice by medium- and high-dose MSC (*p* < 0.05). Low-dose hUC-MSCs significantly elevated serum CXCL1/KC and IL-1β in CS-exposed mice (*p* < 0.05). Our results suggest that high-dose hUC-MSCs reduced pulmonary inflammation and had antiapoptotic effects in acute pulmonary inflammation.

## Introduction

Cigarette smoke (CS) contains numerous toxic chemical compounds and carcinogens (Nyunoya et al., 2014). The median survival age is 85 years in never smokers, 80 years in non-daily smokers, and 75 years in daily smokers (Inoue-Choi et al., 2019). In addition, the mortality risk in non-daily smokers is 72% higher than that in never smokers. CS inhalation has been reported to activate lung epithelial cell inflammation, cause DNA damage, and induce cell death (Nyunoya et al., 2014). After short-term exposure to CS for 5 days, the trachea and diaphragm of mice were infiltrated by inflammatory cells (Martins et al., 2017). Long-term CS exposure increases the risks of chronic obstructive pulmonary disease, lung cancer, and premature death (Nyunoya et al., 2014). However, the treatment strategies currently used for acute pulmonary inflammation, which include bronchodilators, anti-inflammatory drugs, oxygen, and mechanical ventilation, are only supportive therapies in clinical settings (Levy and Serhan, 2014; Thompson et al., 2017; Crisafulli et al., 2018). Because of the limited number of effective therapies, a novel therapy, for example, stem cell therapy, is required to resolve acute pulmonary inflammation cases by CS exposure.

The role of mesenchymal stem cells (MSCs) in immunoregulation has been investigated for clinical purposes. A decreased level of angiopoietin-2 after the administration of a single dose of MSC has been reported in moderate to severe acute respiratory distress syndrome (Matthay et al., 2019). Following two doses of MSC and lung volume reduction surgery, cluster of differentiation (CD)-31 and CD3^+^ T cells increased in severe emphysema (Stolk et al., 2016). According to the above mentioned descriptions, MSCs have potential for lung disease treatment. However, the interaction between MSCs and lung disease should be investigated in additional studies.

MSCs are derived from the bone marrow, umbilical cord, and adipose tissue. Human umbilical cord-derived MSCs (hUC-MSCs) have a more steady doubling time, rarely produce teratomas, and possess a higher differentiation capability than bone marrow-derived MSCs. Reduced expression of major histocompatibility complex classes I and II has been observed to reduce immunogenicity in hUC-MSCs (Li et al., 2015). Recently, the antifibrosis and immunomodulation properties of hUC-MSCs through prostaglandin E2 have been demonstrated (Wen et al., 2018). hUC-MSCs reduced reactive oxygen species and increased anti-inflammatory responses in acute lung injury (ALI) *in vivo* (Liu et al., 2016; Curley et al., 2017). hUC-MSCs also mitigated inflammation and increased the percentage of regulatory T cells in ovalbumin-induced murine models (Kang et al., 2017).

Promoting cytokine and chemokine production can induce the homing process of MSCs in damaged tissue (Zachar et al., 2016). Most MSCs can enter the lung through intravenous infusion (Fischer et al., 2009). MSC migration to the lung may be possible due to the cell size and guidance by adhesion molecules. Therefore, MSCs have a first-pass effect on lungs because of their cellular characteristics. MSCs have been reported to attenuate lung fibrosis by decreasing pro-inflammatory cytokine production and collagen deposition (Su et al., 2018). It also decreased perinatal inflammation and restored lung development in hyperoxia-induced bronchopulmonary dysplasia (Chou et al., 2016). In allergic diseases, MSCs regulated airway remodeling and reduced inflammatory cells in rhinitis and asthma mouse models (Dai et al., 2017; Isik et al., 2017). However, the effects of hUC-MSCs on CS-induced acute pulmonary inflammation still have to be elucidated. The present study investigated the effects of hUC-MSCs on lung injury and inflammation in the acute CS-induced pulmonary inflammation mouse model.

## Materials and Methods

### Animal

Male C57BL/6JNarl mice (8 weeks, 20–25 *g*) were obtained from the National Laboratory Animal Center (Taipei, Taiwan). A constant temperature (22 ± 2°C) and relative humidity (55 ± 10%) and a 12 h:12 h light:dark cycle were maintained throughout the study. The mice were housed in plastic cages and were administered Lab Diet 5001 (PMI Nutrition International, St. Louis, MO, United States), and they had *ad libitum* access to water during acclimatization. The animal experiments were performed in compliance with the Animal and Ethics Review Committee of the Laboratory Animal Center at Taipei Medical University (Taipei, Taiwan; LAC-2017-0231).

### Establishment of the Cigarette Smoke-Induced Acute Pulmonary Inflammation Model

**FIGURE 1:**
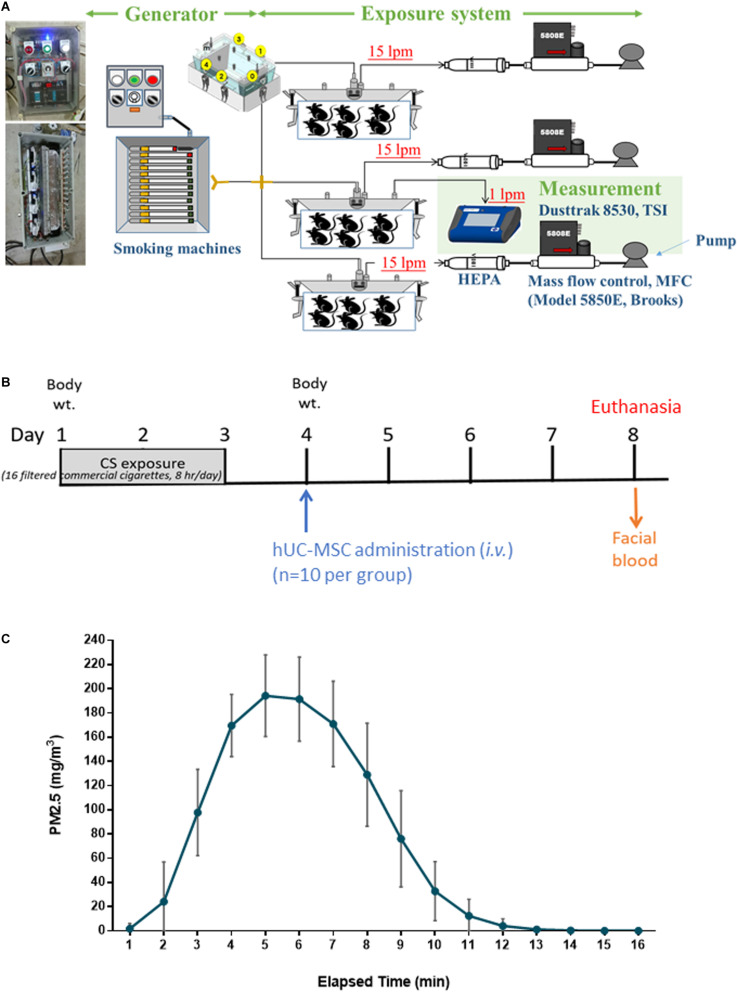
Experiment design for **(A)** whole-body exposure system for cigarette smoke (CS), **(B)** human umbilical cord-derived mesenchymal stem cell (hUC-MSC) treatment on models exposed to acute CS exposure *in vivo* (*n* = 10 per group), and **(C)** distribution of PM_2_._5_ in mass concentrations during CS exposure. Mainstream smoke from the commercial cigarette combustion (16 cigarettes/8 h/day) was introduced into the whole-body exposure chambers. The mice in the exposure chambers were exposed to CS for 3 days. CS-exposed mice were intravenously administered a single dose of hUC-MSCs (CS + MSC-L: 3 × 10^6^ cells/kg, CS + MSC-M: 1 × 10^7^ cells/kg, and CS + MSC-H: 3 × 10^7^ cells/kg). All of the mice were under euthanasia on Day 8 after hUC-MSC administration.

The CS exposure system had three major components, namely, a CS generator, a whole-body exposure system, and measurement devices, as illustrated in Figure 1A. The CS generator included a controller and a smoking machine for CS combustion. There were 16 slots equipped with electronic lighting control in the generator. The commercial cigarette (Mevius, Original, Japan) was continuous combustion without air suction after lighting. Clean air filtered with a high-efficiency particulate air (HEPA) filter was introduced into the CS generator. Mainstream smoke from the combustion of one commercial cigarette was then introduced into the whole-body exposure system (TECNIPLAST, Italy) at the flow rate of 15 liters per minute (lpm) for a time course of 16 cigarettes/8 h/day for 3 days. The mass concentration of particulate matter less than 2.5 μm (PM_2_._5_) was determined using a DustTrak monitor (8530, TSI, Minnesota, United States). The PM_2_._5_ emitted from cigarette combustion was measured four times (1 time/cigarette) per day.

### Human Umbilical Cord-Derived Mesenchymal Stem Cell Preparation

Human umbilical cord-derived mesenchymal stem cells were obtained from Meridigen Biotech Co., Ltd. (Taipei, Taiwan). The cells used in the present study were followed by the International Society for Cellular Therapy Guidelines. Umbilical cord tissue was harvested under sterile conditions and digested with collagenase (SERVA, Heidelberg, Germany) for 120 min in a 37°C incubator. Digestion was terminated in α-minimal essential culture medium (Invitrogen, Waltham, MA, United States) supplemented with 18% fetal bovine serum (Invitrogen), 4 ng/ml basic fibroblast growth factor (Peprotech, Rocky Hill, NJ, United States), and 50 mg/ml gentamicin. The cells were subsequently incubated in a humidified incubator with 5% CO_2_ at 37°C for 3 days, at which point the culture medium was replenished, and the non-adherent cells were removed. hUC-MSCs were passaged once reached 80–90% confluence to the sixth generation. For long-term storage, hUC-MSCs were suspended in CryoStor CS10 (STEMCELL Technologies, Vancouver, BC, Canada) and cryopreserved in a vapor phase liquid nitrogen tank. This study was approved by the Ethics Committee of the National Cheng Kung University Hospital Institutional Review Board (Tainan, Taiwan). All subjects received written and oral information prior to inclusion and provided informed consent. All study processes were carried out in accordance with the approved study protocol (A-BR-104-045).

### Human Umbilical Cord-Derived Mesenchymal Stem Cell Administration

The experiment design is depicted in Figure 1B. The total number of 50 mice was used in this study and the 10 mice in each experimental group. On Day 1, the mice were exposed to CS for 3 days. Control mice were exposed to CS-free HEPA-filtered room air (RA). On Day 4, CS-exposed mice were intravenously administered (*via* tail vein) a single dose of hUC-MSCs (CS + MSC-L: 3 × 10^6^ cells/kg, CS + MSC-M: 1 × 10^7^ cells/kg, and CS + MSC-H: 3 × 10^7^ cells/kg) provided by Meridigen Biotech Co., Ltd., in clinical grade normal saline containing 2% clinical grade human serum albumin and 16.7% clinical grade CS10. Control mice were given the same volume of vehicle alone. On Day 8, all mice were anesthetized [intraperitoneally (i.p.)] under Zoletil (Virbac, Taiwan) and xylazine, followed by CO_2_ euthanasia. The facial blood, bronchoalveolar lavage fluid (BALF), and whole lungs were collected (*n* = 10 per group). The BALF was collected by washing the lungs three times with 0.5 ml of phosphate-buffered saline (PBS). The BALF samples were centrifuged at 200G for 10 min at 4°C. The supernatant and pellets were collected for biochemical and hematology analysis, respectively. The submandibular method was used for blood collection. The blood samples were stored at room temperature for an hour following centrifugation at 200G under 4°C for 10 min.

### Body Weight

The mouse body weight was measured on Day 1 (the day before CS exposure), Day 4 (the day before hUC-MSC administration), and Day 8.

### Cell Count Analysis in Bronchoalveolar Lavage Fluid

The BALF pellets were resuspended with 40 μl of PBS for hematology analysis (ProCyte Dx; IDEXX Laboratories; Westbrook, ME, United States). The total cell, neutrophil, lymphocyte, monocyte, and eosinophil counts were measured using a hematology analyzer. For the differential cell analysis, the data were presented as percentages (%) of the total cell number.

### Protein Extraction in Lung Tissues

Lung tissues were homogenized in 490 μl of lysis reagent (Sigma-Aldrich, Inc., St. Louis, MO, United States) with 5 μl of protease inhibitor (Geno Technology Inc., St. Louis, MO, United States) and 5 μl of ethylenediaminetetraacetic acid using a homogenizer (Minilys personal homogenizer; Bertin; Rockville, MD, United States), according to the manufacturers’ instructions.

### Enzyme-Linked Immunosorbent Assay

Enzyme-linked immunosorbent assay (ELISA) was used to investigate the tumor necrosis factor (TNF)-α (Invitrogen, Waltham, MA, United States), chemokine (CXC motif) ligand 1/keratinocyte chemoattractant (CXCL1/KC; R&D Systems Inc., Minneapolis, MN, United States), and interleukin (IL)-1β (Invitrogen) in the BALF and serum samples. TNF-α, KC, IL-1β, matrix metallopeptidase (MMP)-9 (R&D Systems), and caspase-3 levels (Elabscience, Houston, Texas, United States) in lung lysates were determined. All of the ELISA kits were conducted following the manufacturers’ instructions. The levels of these markers determined in lung tissues were adjusted based on the total protein in lung lysates. The detailed information of these ELSA kits was shown in Supplementary Table S1.

### Statistical Analysis

Ten mice in each group were included in each analysis. The Shapiro–Wilk’s test was used to examine the normality. The data are expressed as mean ± standard deviation (SD). The data from repeated measurement were compared using the paired *t* test. Comparisons within multiple groups were performed through analysis of variance (ANOVA) with Fisher’s *post hoc* test. Statistical analyses were conducted using GraphPad ver. 7 (San Diego, CA, United States) for Microsoft Windows. The significance criterion was set to *p* < 0.05.

## Results

### PM_2_._5_ Mass Concentration During Cigarette Smoke Exposure

As presented in Figure 1C, PM_2_._5_ was generated at an average of 108.7 ± 72.8 mg/m^3^ during the first 10 min of combustion. The concentration peaked at 194.2 ± 33.7 mg/m^3^ approximately between 5 and 6 min and started to decline afterward.

### Body Weight

**FIGURE 2:**
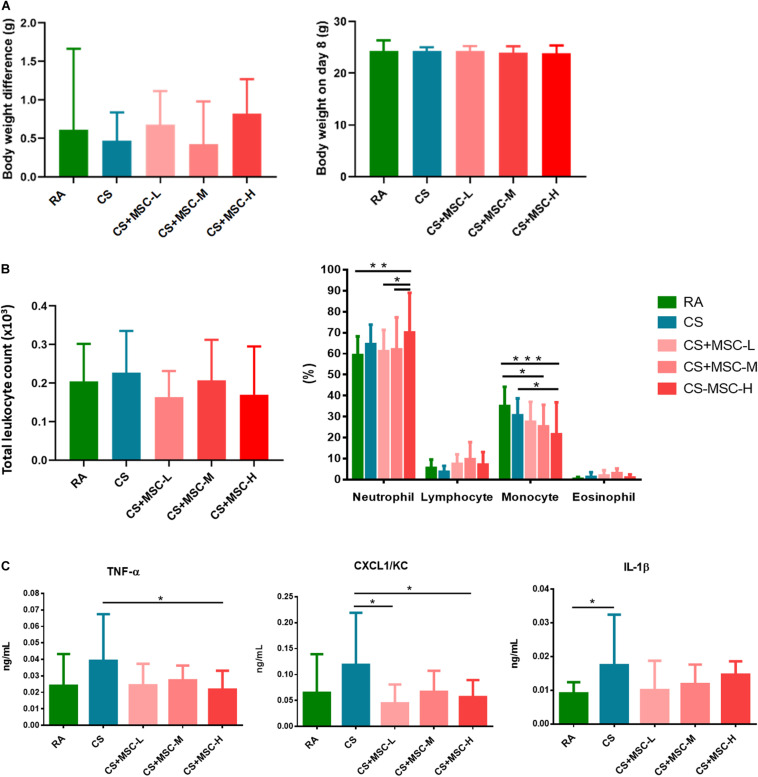
**(A)** Body weight change before and after human umbilical cord-derived mesenchymal stem cell (hUC-MSC) administration; **(B)** Cell counts in the bronchoalveolar lavage fluid (BALF) for the room air (RA) group, cigarette smoke (CS) group, CS + MSC-L (CS with 3 × 10^6^ cells/kg MSC), CS + MSC-M (CS with 1 × 10^7^ cells/kg MSC), and CS + MSC-H (CS with 3 × 10^7^ cells/kg MSC); and **(C)** tumor necrosis factor (TNF)-α, (CXC motif) ligand 1/keratinocyte chemoattractant (CXCL1/KC), and interleukin (IL)-1β in the BALF for the room air (RA) group, CS group, CS + MSC-L (CS with 3 × 10^6^ cells/kg MSC) group, CS + MSC-M (CS with 1 × 10^7^ cells/kg MSC) group, and CS + MSC-H (CS with 3 × 10^7^ cells/kg MSC) group. The neutrophils were significantly increased in the CS + MSC-H group (70.05 ± 18.99%) than those in the RA (59.26 ± 8.98%), the CS + MSC-L (61.12 ± 10.17%), and the CS + MSC-M (62.05 ± 15.24%) groups. A significant decrease in monocytes (%) was determined in the CS + MSC-H (21.52 ± 15.25%) when compared with those in the RA (34.98 ± 9.23%) and CS (30.63 ± 8.05%) groups. TNF-α was significantly decreased by CS + MSC-H (0.022 ± 0.011 ng/ml) when compared with that in the CS group (0.039 ± 0.028 ng/ml). The levels of CXCL1/KC in the CS + MSC-L (0.045 ± 0.036 ng/ml) and CS + MSC-H (0.056 ± 0.033 ng/ml) groups were significantly reduced compared with that in the CS group (0.119 ± 0.100 ng/ml). *n* = 10 in each group. Data were presented as mean ± SD. The body weight difference was determined by the paired *t* test. The cell counts and cytokines in BALF of mice were tested by ANOVA with Fisher’s *post hoc* test. *p < 0.05; **p < 0.01; and ***p < 0.001.

Figure 2A presents the body weight difference and the body weight measured on Day 8 after hUC-MSC administration. The body weight difference was determined between the mice exposed to RA, CS, and CS followed by hUC-MSC administration. There was no statistical difference between the groups.

### Human Umbilical Cord-Derived Mesenchymal Stem Cells Regulated Neutrophils and Monocytes in Bronchoalveolar Lavage Fluid

The total cell counts and differential cell counts (%) in the BALF were determined, and the results are shown in Figure 2B. There were no significant differences of total cell counts between the groups. We observed a significant increase in neutrophil (%) production after high-dose hUC-MSC administration (3 × 10^7^ cells/kg; *p* < 0.05). Conversely, monocytes significantly decreased after medium- (1 × 10^7^ cells/kg), and high-dose (3 × 10^7^ cells/kg) hUC-MSC administration (*p* < 0.05). No significant difference was observed in lymphocytes and eosinophils among the groups.

### Human Umbilical Cord-Derived Mesenchymal Stem Cells Decreased the Levels of Tumor Necrosis Factor-α, (CXC Motif) Ligand 1/Keratinocyte Chemoattractant, and Interleukin-1β in Bronchoalveolar Lavage Fluid

After hUC-MSC administration, TNF-α, CXCL1/KC, and IL-1β levels in the BALF were determined, as illustrated in Figure 2C. IL-1β in BALF was significantly increased in the CS group as compared with that in the RA group, indicating that the mice were induced acute lung inflammation after 3 days of CS exposure. We observed that high-dose hUC-MSC administration (3 × 10^7^ cells/kg) significantly reduced the level of TNF-α (*p* < 0.05). Low- (3 × 10^6^ cells/kg) and high-dose (3 × 10^7^ cells/kg) hUC-MSC administration also significantly decreased CXCL1/KC (*p* < 0.05). No significant decrease in IL-1β was caused by hUC-MSCs after CS exposure.

### Human Umbilical Cord-Derived Mesenchymal Stem Cells Reduced the Levels of Tumor Necrosis Factor-α, (CXC Motif) Ligand 1/Keratinocyte Chemoattractant, Interleukin-1β, Matrix Metallopeptidase-9, and Caspase-3 in Lung Lysates

**FIGURE 3:**
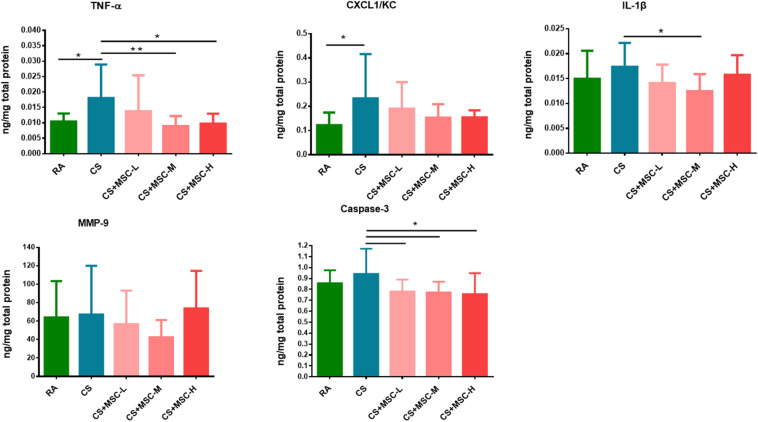
Tumor necrosis factor (TNF)-α, (CXC motif) ligand 1/keratinocyte chemoattractant (CXCL1/KC), interleukin (IL)-1β, matrix metallopeptidase (MMP)-9, and caspase-3 in lung tissue samples. The levels of TNF-α in the CS + MSC-M (0.009 ± 0.003 ng/mg) and CS + MSC-H (0.010 ± 0.003 ng/mg) groups were significantly decreased compared with that in the cigarette smoke (CS) group (0.018 ± 0.011 ng/mg). Decreased IL-1β in the CS + MSC-M (0.012 ± 0.003 ng/mg) group was observed than that in the CS group (0.017 ± 0.0048 ng/mg). The caspase-3 levels were significantly reduced by human umbilical cord-derived mesenchymal stem cells (hUC-MSCs; 0.781 ± 0.109, 0.772 ± 0.099, and 0.758 ± 0.191 ng/mg in the low-, medium-, and high-dose hUC-MSCs groups, respectively) than that in the CS group (0.942 ± 0.229 ng/mg). *n* = 10 in each group. Data were presented as mean ± SD. The cytokines in lung tissue of mice were assessed by ANOVA with Fisher’s *post hoc* test. *p < 0.05; **p < 0.01.

The levels of TNF-α, CXCL1/KC, IL-1β, and MMP-9 in lung lysates were determined. As displayed in Figure 3, we observed that TNF-α and CXCL1/KC in lung were considerably higher in the CS group compared with those in the RA group, suggesting acute pulmonary inflammation was induced in the mice of the CS group. The TNF-α level was significantly reduced after medium- (1 × 10^7^ cells/kg) and high-dose (3 × 10^7^ cells/kg) hUC-MSC administration (*p* < 0.05). No significant difference was observed in CXCL1/KC production after hUC-MSC administration. Medium-dose (1 × 10^7^ cells/kg) hUC-MSC administration significantly reduced IL-1β in lung tissues (*p* < 0.05). There were no significant differences in MMP-9 between the groups. The hUC-MSCs exhibited a significant mitigating effect on apoptosis by reducing caspase-3 (*p* < 0.05).

### Human Umbilical Cord-Derived Mesenchymal Stem Cells Changed the Serum Levels of Tumor Necrosis Factor-α, (CXC Motif) Ligand 1/Keratinocyte Chemoattractant, and Interleukin-1β

**FIGURE 4:**
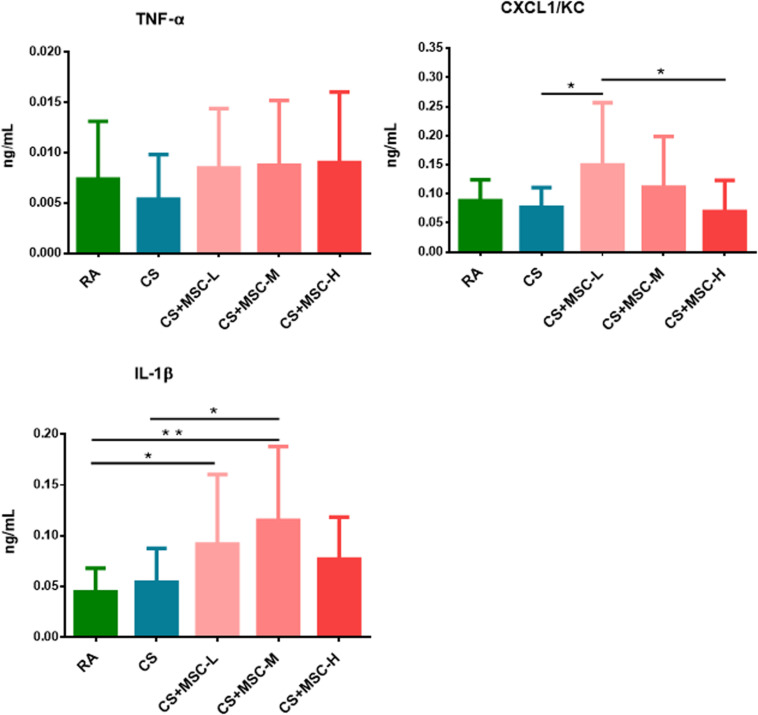
Serum tumor necrosis factor (TNF)-α, (CXC motif) ligand 1/keratinocyte chemoattractant (CXCL1/KC), and interleukin (IL)-1β changed after human umbilical cord-derived mesenchymal stem cell (hUC-MSC) administration. We observed that the level of CXCL1/KC in the CS + MSC-L group (0.150 ± 0.107 ng/ml) was significantly increased than those in the cigarette smoke (CS; 0.076 ± 0.034 ng/ml) and CS + MSC-H (0.069 ± 0.054 ng/ml) groups. Increase in IL-1β was observed in the CS + MSC-L (0.092 ± 0.069 ng/ml) and CS + MSC-M (0.115 ± 0.073 ng/ml) groups when compared with that in the RA group (0.044 ± 0.023 ng/ml), whereas IL-1β was significantly increased in the CS + MSC-M group (0.115 ± 0.073 ng/ml) than that in the CS group (0.054 ± 0.034 ng/ml). *n* = 10 in each group. Data were presented as mean ± SD. The ANOVA with Fisher’s *post hoc* test were conducted to test the serum level of cytokines in mice. *p < 0.05; **p < 0.01.

The serum levels of TNF-α, CXCL1/KC, and IL-1β were determined, and the results are shown in Figure 4. TNF-α levels showed no significant difference among the groups. Increased CXCL1/KC levels were observed after low-dose (3 × 10^6^ cells/kg) hUC-MSC administration compared with after CS and high-dose (3 × 10^7^ cells/kg) hUC-MSC administration (*p* < 0.05). The administration of low (3 × 10^6^ cells/kg) and medium (1 × 10^7^ cells/kg) doses of hUC-MSCs significantly increased IL-1β levels compared with RA (*p* < 0.05), whereas IL-1β was increased by the medium dose of hUC-MSCs when compared with that of the CS group (*p* < 0.05).

## Discussion

Human umbilical cord-derived mesenchymal stem cells have been reported to regulate inflammation and protect host cells from apoptosis (Li et al., 2015). In our study, we investigated the effects of hUC-MSCs on CS-induced acute pulmonary inflammation. The main results were summarized in Supplementary Table S2. We observed that hUC-MSCs mitigated pro-inflammatory cytokine production of BALF and lung tissue in the acute CS-induced pulmonary inflammation mouse model, especially at high-dose administration. hUC-MSCs exerted antiapoptotic effects by reducing the caspase-3 production in the lungs of the mice. Our results suggest that high-dose of hUC-MSCs could decrease inflammatory responses during acute pulmonary inflammation *in vivo.*

The CS exposure system in the present study was used to determine the adverse health effects of short-term CS exposure in the lung of mice. Previous studies have reported that mice exposed to CS for 4 days showed acute lung inflammation (Nemmar et al., 2012). In the present study, mice were exposed to mainstream CS for 3 days through a whole-body exposure chamber. This exposure system and approach reduced the stress caused by CS exposure. The mice were exposed to PM_2_._5_ at an average of 108.7 ± 72.8 mg/m^3^ during the study period. Consistently, in previous reports, mice were exposed to CS at an average of 90–100 mg/m^3^ (Chen et al., 2005; Carter and Misra, 2010; Schweitzer et al., 2011; Siggins et al., 2014).

A previous report revealed that the majority of intravenously injected MSCs could migrate into the lungs within 24 h (Eggenhofer et al., 2012). Compared with bone marrow- and gingival tissue-derived MSCs, hUC-MSCs have been determined to display the highest immunomodulatory ability (Li et al., 2018). Doses of hUC-MSC between 1 × 10^6^ and 4 × 10^6^ cells/ml have been deemed effective in a previous study (Chen et al., 2005; Carter and Misra, 2010; Lin et al., 2017). For example, the hUC-MSC concentration of 3 × 10^6^ cells/ml was administered in lung disease (Zhang et al., 2018). However, the role of hUC-MSCs in acute pulmonary inflammation has not been fully elucidated. Therefore, we investigated the effects of hUC-MSCs [intravenously (i.v.)] on the acute CS-induced lung inflammation mouse model. First, we observed body weight change after hUC-MSC administration. No significant difference was found among the groups. A previous study indicated that the body weight and appetite of mice were reduced after 4 days of CS exposure (Chen et al., 2005). Another study described that the body weight of mice under chronic CS exposure increased after stem cell administration (Schweitzer et al., 2011). In the present study, the body weight slightly changed but at a non-significant statistical level, which could result from the short-term CS exposure.

In this study, we observed a significant increase in neutrophil production and a reduction of monocytes in the BALF due to hUC-MSCs. Previous studies have reported that an increase in neutrophil phagocytic activity by MSC promoted bacteria clearance in septic mouse model (Hall et al., 2013). In contrast, neutrophil counts in the BALF significantly decreased after hUC-MSC administration in Balb/c mice with lipopolysaccharide (LPS)-induced ALI (Zhu et al., 2017). MSCs decreased neutrophils, macrophages, and lymphocytes in the BALF after subacute (7 weeks) CS exposure in Sprague Dawley rats (Song et al., 2014). Both models of acute and subacute CS exposure exhibited a reduction of neutrophils in the BALF after MSC administration. The difference may be due to the various sources of MSCs as well as the tested animal models. Monocyte counts in the BALF of mice with endotoxin-induced ALI decreased after MSC administration (Hao et al., 2015). Consistently, our findings also revealed a significant reduction of monocytes in the BALF of the mice after hUC-MSC administration. The interaction between monocytes and MSCs has been reported previously. The microvesicles released by MSCs may enhance the monocyte phagocytosis and decrease inflammatory cytokine secretion (Monsel et al., 2015). Monocytes also differentiate into macrophages based on local growth factors and cytokines (Shi and Pamer, 2011). Our observation suggests that diverse regulating mechanisms of hUC-MSCs may be involved in alleviating acute pulmonary inflammation.

In this study, we observed a decrease in inflammatory TNF-α and CXCL1/KC in the BALF by hUC-MSC, and the TNF-α and IL-1β were reduced in lung tissue of the mice after hUC-MSC administration. In line with other studies, the levels of TNF-α and cytokine-induced neutrophil chemoattractant-1 in the BALF of rats were reduced by MSCs at 4 and 12 h after gastric aspiration-induced lung injury (Zhou et al., 2016). TNF-α, IL-6, and macrophage inflammatory protein-2 in the BALF decreased after MSC intervention in a rat model of ventilator-induced lung injury (Lai et al., 2015). Most inflammatory mediators (IL-1β, IL-6, and interferon-γ) in mouse lungs were mitigated within 7 days of MSC treatment in ALI (Liu et al., 2015). These observations indicate that MSCs may resolve localized pulmonary inflammation. Previous study showed that the IL-6, IL-8, and granulocyte-macrophage colony-stimulating factor (GM-CSF) secreted by MSC enhanced the migration of neutrophil to the injury site (Joel et al., 2019). Our results suggested that the increase in neutrophils and reduction of cytokines (TNF-α, CXCL1/KC, and IL-1β) in BALF and lung samples could be involved in different pathways and expressed at different time points after hUC-MSC administration. Notably, in this study, we observed that the levels of caspase-3 decreased after hUC-MSC administration. The results indicate that hUC-MSCs may be involved in the regulation of apoptosis, which is also supported by previous *in vivo* and *in vitro* studies (Jiang et al., 2015; Li et al., 2017). hUC-MSCs may regulate the inflammatory responses and may exhibit antiapoptotic effects in the acute pulmonary inflammation model.

Furthermore, we discovered that the serum levels of CXCL1/KC significantly increased by low-dose human umbilical cord-derived mesenchymal stem cells. The levels of IL-1β in serum were increased after low and medium doses of hUC-MSC administration. Intravenous infusion of MSCs in mice was proven to enhance the systemic inflammatory response [G-CSF, IL-6, CXCL1, and CC motif chemokine ligand (CCL)-2] within 2 h, which was followed by a reduction of immune reactivity (Hoogduijn et al., 2013). By contrast, previous studies have demonstrated that the MSCs significantly reduced systemic cytokines and chemokines (IL-6, IL-1b, IL-10, KC, and CCL5) after 28 h of administration in the sepsis-associated inflammation mouse model (Mei et al., 2010). MSCs decreased mouse IL-6 production in serum after LPS-induced acute pulmonary inflammation (Khedoe et al., 2017). We observed that hUC-MSCs increased systemic inflammatory responses after 4 days of administration. This observation may be associated with hUC-MSC-regulated neutrophil infiltration in the lungs. Neutrophils can induce IL-1β production (Iula et al., 2018). For example, neutrophil elastase can cleave the pro-isoform of IL-1β in endothelial cells to release the active form into the extracellular space (Alfaidi et al., 2015). Moreover, IL-1β has been shown to induce CXCL1 and CXCL2 for recruiting neutrophil infiltration in the inflammatory sites (Biondo et al., 2014). Therefore, the increase in circulating IL-1β and CXCL1/KC may be associated with neutrophil infiltration in the lungs of mice.

Although we determined the effects of hUC-MSCs on acute pulmonary inflammation *in vivo*, our study has some limitations. We observed the effects of hUC-MSCs on the acute pulmonary inflammation model; however, the regulation of hUC-MSCs in the chronic CS exposure model remains unclear. hUC-MSCs were administered for 4 days, but the chronic effects of hUC-MSCs on lungs have not been determined in the present study. We noted that high-dose hUC-MSCs reduced lung inflammation but resulted in neutrophil infiltration. Additional studies are required to better understand this phenomenon. The quantity of oxidative stress and mechanistic experiments (Western blot, immunohistochemistry) will be used to investigate the role of hUC-MSC in CS-induced pulmonary inflammation *in vivo*. The chronic CS-induced pulmonary inflammation mouse model will be used in future works.

## Conclusion

The present study adds to the recent research by demonstrating that hUC-MSCs mitigate pulmonary inflammation in the acute CS-induced pulmonary inflammation mouse model. Moreover, the administration of hUC-MSCs reduces apoptosis in the lungs. Our findings demonstrate that hUC-MSCs can attenuate pulmonary inflammation and may exhibit antiapoptotic effects in the short-term CS-exposed pulmonary inflammation model. hUC-MSCs may be a novel therapeutic strategy for acute pulmonary inflammation disease.

## Data Availability Statement

The raw data supporting the conclusions of this article will be made available by the authors, without undue reservation.

## Ethics Statement

The studies involving human participants were reviewed and approved by Ethics Committee of the National Cheng Kung University Hospital Institutional Review Board. The patients/participants provided their written informed consent to participate in this study. The animal study was reviewed and approved by Animal and Ethics Review Committee of the Laboratory Animal Center at Taipei Medical University. Written informed consent was obtained from the owners for the participation of their animals in this study.

## Author Contributions

H-CC and X-YC contributed to the completion of interpretation of the data and the manuscript. H-CC, WL, and C-WC contributed substantially to the concept, design, interpretation of the data, and completion of the study and the manuscript. Y-YC, C-HC, and Y-CW contributed substantially to the completion of the study. T-CH contributed to the establishment of the cigarette smoke generation system and particle measurement. All authors contributed to critically revising the manuscript for important intellectual content. All authors have read and approved the final manuscript.

## Conflict of Interest

The authors declare that the research was conducted in the absence of any commercial or financial relationships that could be construed as a potential conflict of interest.
