# Supplementary material for: Effects of Human Umbilical Cord-Derived Mesenchymal Stem Cells on the Acute Cigarette Smoke-Induced Pulmonary Inflammation Model
Source: Front Physiol. 2020 Aug 12;11:962. doi: 10.3389/fphys.2020.00962 (PMC7434987; doi:10.3389/fphys.2020.00962)
Supplement: Supplementary file 1 [file Data_Sheet_1.pdf]

Supplementary Information

Effects of human umbilical cord-derived mesenchymal stem cells on the acute cigarette smoke-induced pulmonary inflammation model

Xiao-Yue Chen[1], MSc., Yi-Ying Chen[1], MSc., Willie Lin[2], PhD., Chia-Wen Chien[2], PhD., Chien-Han Chen[2], MSc., Yu-Chieh Wen[2], MSc., Ta-Chih Hsiao[3], PhD., Hsiao-Chi Chuang[1,4,5]*, PhD.

[1]School of Respiratory Therapy, College of Medicine, Taipei Medical University, Taipei, Taiwan

[2]Meridigen Biotech Co., Ltd., Taipei, Taiwan

[3]Graduate Institute of Environmental Engineering, National Taiwan University, Taipei, Taiwan

[4]Cell Physiology and Molecular Image Research Center, Wan Fang Hospital, Taipei Medical University, Taipei, Taiwan

[5]Division of Pulmonary Medicine, Department of Internal Medicine, Shuang Ho Hospital, Taipei Medical University, New Taipei City, Taiwan

Supplementary Information

Table S1. The product information of ELISA kits used in the present study.

ELISA Kit	brand	Production No.	Lot No.	Assay range	Sensitivity
TNF-α	Thermo Fisher Scientific	88-7324-88	4347661	8-1,000 pg/mL	8 pg/mL
CXCL1/KC	R&D Systems	DY453	P137636	16-1,000 pg/mL.	
IL-1β	Thermo Fisher Scientific	88-7013-88	4341152	8-1,000 pg/mL	8 pg/mL
MMP-9	R&D Systems	DY6718	P146772	78-5000 pg/mL	
Caspase-3	Elabscience	E-EL-M0238	AK0017NOV6028	0.16-10 ng/mL	0.10 ng/mL

Supplementary Information

Table S2. Summnary of main results in the present study

	**BALF**				**Lung lysate**				**Serum**			
	**RA***	**CS+MSC-L^{+}**	**CS+MSC-M^{+}**	**CS+MSC-H^{+}**	**RA***	**CS+MSC-L^{+}**	**CS+MSC-M^{+}**	**CS+MSC-H^{+}**	**RA***	**CS+MSC-L^{+}**	**CS+MSC-M^{+}**	**CS+MSC-H^{+}**
**Total cell count (×10^{3})**	-	-	-	-	**N/A**				**N/A**			
**Neutrophil (%)**	-	-	-	-	**N/A**				**N/A**			
**Lymphocyte (%)**	-	-	-	-	**N/A**				**N/A**			
**Monocyte (%)**	-	-	-	↓	**N/A**				**N/A**			
**Eosinophil (%)**	-	-	-	-	**N/A**				**N/A**			
**TNF-α**	-	-	-	↓	↑	-	↓	↓	-	-	-	-
**CXCL1/KC**	-	↓	-	↓	↑	-	-	-	-	↑	-	-
**IL-1β**	↑	-	-	-	-	-	↓	-	-	-	↑	-
**MMP-9**	**N/A**				-	-	-	-	**N/A**			
**Caspase-3**	**N/A**				-	↓	↓	↓	**N/A**			

*The CS group were compared with the RA group; $^{+}$MSC groups were compared with the CS group. N/A: Not applicable.
